# Raman spectroscopy-based identification of toxoid vaccine products

**DOI:** 10.1038/s41541-018-0088-y

**Published:** 2018-10-04

**Authors:** Anja Silge, Thomas Bocklitz, Bjoern Becker, Walter Matheis, Juergen Popp, Isabelle Bekeredjian-Ding

**Affiliations:** 1 Institute of Physical Chemistry and Abbe Center of Photonics, Friedrich-Schiller University Jena, Helmholtzweg 4, D-07743 Jena, Germany; 2 InfectoGnostics Research Campus Jena, Centre of Applied Research, Philosophenweg 7, D-07743 Jena, Germany; 3 Leibniz Institute of Photonic Technology, Albert-Einstein-Str. 9, D-07745 Jena, Germany; 4 Division of Microbiology, Paul-Ehrlich-Institut, Paul-Ehrlichstr. 51-59, D-63225 Langen, Germany

## Abstract

Vaccines are complex biomedicines. Manufacturing is time consuming and requires a high level of quality control (QC) to guarantee consistent safety and potency. An increasing global demand has led to the need to reduce time and cost of manufacturing. The evolving concepts for QC and the upcoming threat of falsification of biomedicines define a new need for methods that allow the fast and reliable identification of vaccines. Raman spectroscopy is a non-destructive technology already established in QC of classical medicines. We hypothesized that Raman spectroscopy could be used for identification and differentiation of vaccine products. Raman maps obtained from air-dried samples of combination vaccines containing antigens from tetanus, diphtheria and pertussis (DTaP vaccines) were summarized to compile product-specific Raman signatures. Sources of technical variance were emphasized to evaluate the robustness and sensitivity in downstream data analysis. The data management approach corrects for spatial inhomogeneities in the dried sample while offering a proper representation of the original samples inherent chemical signature. Reproducibility of the identification was validated by a leave-one-replicate-out cross-validation. The results highlighted the high specificity and sensitivity of Raman measurements in identifying DTaP vaccine products. The results pave the way for further exploitation of the Raman technology for identification of vaccines in batch release and cases of suspected falsification.

## Quality control: Rapid and reliable vaccine analysis

A light-based identification method offers a fast, reliable and non-destructive method to analyze vaccines. Using vaccines for tetanus, diphtheria, and pertussis, a German research team led by the Paul Ehrlich Institute and University of Jena and Leibniz-IPHT showed that Raman spectroscopy — which identifies substances based on how they scatter laser light — is able to identify distinctive signatures of vaccines. In their experiments, Raman spectroscopy was sensitive enough to detect subtle differences in vaccine formulation, such as the specific combination of vaccine and adjuvant components. However, a data analytics technique was required to correct for sample quality variation caused by their preparation. Raman spectroscopy already sees use in classical medicines, and its application to vaccines could help to reduce the time and cost of quality control while benefitting the unmet need for rapid analysis of vaccine quality and identity.

## Introduction

Due to their size and complexity, the quality of biomedicines cannot be characterized nor controlled by conventional chemical methods. Instead, regulatory requirements for biomedicines include a tightly controlled manufacturing processes and batch release by both the manufacturers and official medicines control laboratories (OMCL). The methods used for product characterization and batch testing are defined in the marketing authorisation and need to be approved by the regulatory authority. The regional pharmacopoieia, e.g., European Pharmacopoieia (Ph. E.), provides the legislative framework for product testing and regulatory bodies such as the European Directorate for Quality of Medicines (EDQM) prequalify methods for these purposes, including the biological standards to be used to obtain comparability. Among the methods established for quality control of classical medicines the so called “non-invasive”, e.g., non-destructive, techniques, such as near-infrared and Raman spectroscopy have been applied for molecular imaging and analytics in process analytical technology (PAT) and are implemented in quality by design (QbD) concepts.^1–5^ Recent technical developments in the field of Raman technology now enable manufacturers to use this technique for analysis of more complex biological products including protein mixtures in bioreactors and cell-based and tissue-engineered products.^6–8^ Raman microspectroscopy is an inelastic light scattering-based method useful for the non-destructive analysis of biochemical samples. It provides a wealth of molecular information on a specimen by the sample’s own inherent vibrational signatures. As the biochemical composition of a sample is mirrored in the Raman spectrum, mathematical methods including analytical modelling translate the physically recorded Raman data into higher level information, which can further be exploited for comparative analyses. The fingerprint-like specificity of the spectral signatures can be utilized to setup a reference database of tested biological products for identification purposes.^9–11^

Vaccines used for prophylaxis of infectious diseases range among the eldest and most complex biomedicines. Today, a remarkable number of them still consist of mixtures of attenuated microorganisms or detoxified virulence factors that induce protective immune memory, i.e., either opsonizing or neutralizing antibody responses. The microbial antigens contained in a vaccine are often poorly immunogenic, such as inactivated toxins and are, therefore, provided together with adjuvants that augment the immune response.^12^ The most common adjuvants are poorly soluble aluminium salt-containing gels that adsorb the antigens, thereby forming a suspension.^13,14^ Furthermore, preservatives and excipients may be present in vaccines as well as traces of chemical substances, which usually are residuals from the manufacturing procedure. Thus, any vaccine consists of a unique mixture of chemically distinct substances.^15^ The final composition is considered to be characteristic for every single vaccine product.

Combination vaccines against tetanus, diphtheria and pertussis have successfully been employed over decades. Their active substances are detoxified tetanus, diphtheria and pertussis toxins and, dependent on the product, other *Bordetella pertussis*-derived antigens. Notably, in the Western hemisphere, use of acellular pertussis vaccines predominates because the former whole-cell pertussis vaccine was less well tolerated. Some vaccines additionally contain Hepatitis B antigens and/or inactivated poliovirus. In this study, the authors used tetanus-diphtheria-acellular pertussis (DTaP) vaccines commercially available on the German market.

DTaP combination vaccines are used for primary immunization and booster vaccination in all age groups. They differ in vaccine antigen content and the adjuvants used, e.g., aluminium hydroxide (Al(OH)_3_) and/or aluminium phosphate (AlPO_4_). As in many countries around the world, in the EU, an important prerequisite for marketing of these vaccines is the governmental batch release granted to the marketing authorization holder. For DTaP vaccines, this implies that vaccines are experimentally tested using standardized assays for confirmation of antigen identity and content as well as potency at intermediate and final product stages. Pass and fail criteria need to be defined, and any confirmed out-of-specification results lead to rejection of the vaccine batch by the manufacturer or the authorities.

There is also a growing need for rapid testing procedures that confirm identity and preserved safety and efficacy of medicinal products after market entry. This new demand arises from the increased awareness on falsification of medicines, which also affects biomedicines such as monoclonal antibodies^16^ and potentially vaccines. The development of methods for rapid verification of vaccine identity, antigen concentration, integrity and potency represent a significant requirement in this context.

Raman spectroscopy has already been applied for identification of falsified classical medicines.^17^ In this study, we investigated the potential of Raman spectroscopy for identification and differentiation of DTaP vaccines. The study is driven by a so far unmet need for rapid and reliable methods that support OMCL decision making on product identity and quality. The Raman spectroscopy is a non-invasive technology that provides rapid information on easily accessible product characteristics. Since Raman spectra contain the complete information on the Raman-active chemical content we hypothesized that Raman spectra obtained from vaccine products should be specific for the products and could be used for product identification.

## Results

### Raman spectroscopic analysis of vaccine samples

In the present study, we hypothesized that Raman microspectroscopy could be used to rapidly identify and distinguish vaccine products. Our working hypothesis was that due to differences in formulations vaccine products of the same vaccine type would still differ in their Raman spectra because spectra arising from the multitude of components and excipients would form a unique combined Raman spectral fingerprint that could be used as a product identifier.

**Fig. 1:**
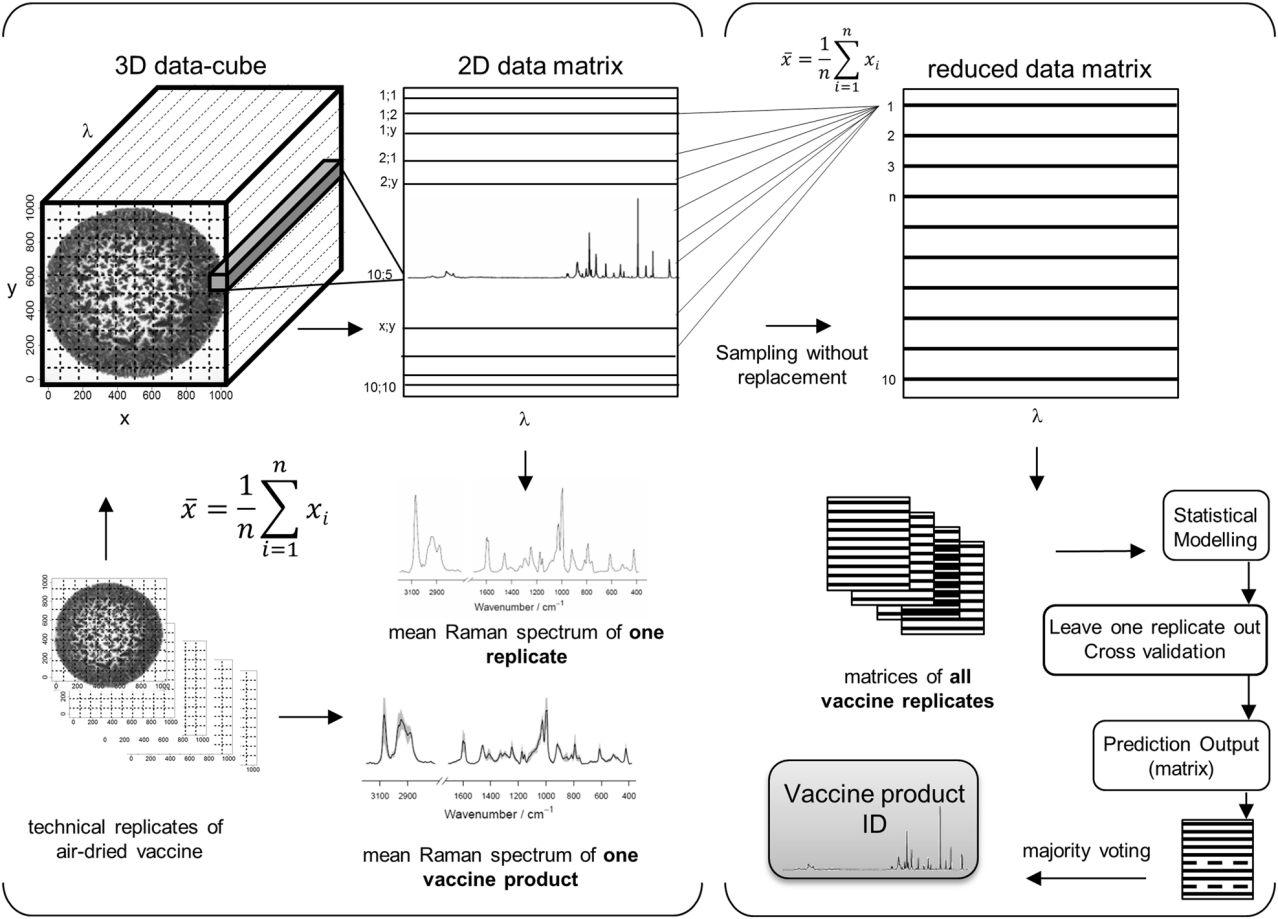
Principle of vaccine Raman spectral data management. Left: Arrangement and output of Raman measurements of an air-dried vaccine droplet is visualized. The microscopic image in the 3D data cube shows the air-dried vaccine sample dTaP-IPV_1_. The overlaid grid visualizes the arranged sample raster of the Raman spectroscopic mapping approach. The hyperspectral data cube is reoriented into a 2D data matrix. To obtain the Raman spectral signature of a distinct vaccine product, the data matrices of the respective replicates were averaged. Right: The processing for the statistical modelling is sketched. The 2D data table of each replicate was reduced from the 100 original spectra to 10 representative spectra. Ten randomly chosen raster points were successive picked without replacement and averaged. The matrices of all vaccine replicates were applied for statistical modelling and cross-validation. The prediction result for one replicate was assembled by the prediction result of its ten spectra. If the prediction result was not uniform for all spectra of one replicate, the prediction output was based on majority rule voting to identify the vaccine product specification

**Fig. 2:**
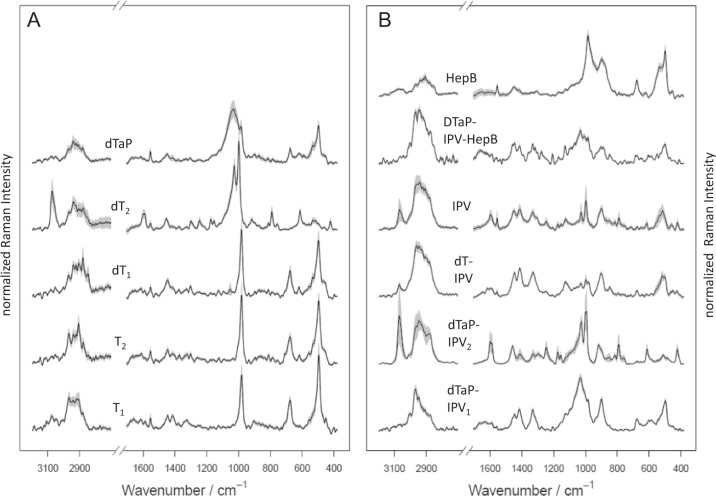
Mean Raman spectra of the investigated vaccine products. Mean spectra present the average of the *n* = 1000 (T_1_), *n* = 1000 (T_2_), *n* = 1000 (dT_1_), *n* = 2100 (dT_2_), *n* = 1100 (IPV), *n* = 1000 (dTaP), *n* = 1100 (dTaP-IPV_1_), *n* = 1100 (dTaP-IPV_2_), *n* = 1000 (dT-IPV), *n* = 1000 (DTaP-IPV-HepB), *n* = 1000 (HepB) measured single Raman spectra. The Raman spectra were collected by the mapping based measurements of technical replicates (air-dried vaccine droplets). The mean spectra visualize the averaged Raman signature of the indicated vaccine products, including the Raman data of the measured replicates with varying drying times. The standard deviations of the means are visualized by the grey shades around each spectrum. All spectra are normalized for an effective comparison and shifted vertically for visualization

For standardization and to obtain optimal Raman spectral information the vaccine suspensions were dried before measurements. A microscope image of an air-dried vaccine sample (dTaP-IPV) is depicted in the 3D data cube in the left part of Fig. 1. The overlaid grid visualizes the arranged sample raster for the mapping. To obtain a comprehensive Raman spectral signature of a unique vaccine product, spectra of all pixels were sequentially arranged in a 2D data matrix. In a first step, the 2D data matrices derived from the replicate measurements were summarized simply by averaging. Figure 2 depicts the averaged Raman spectra derived from the mapped replicates of each vaccine product. The mean spectra reveal that each vaccine may have a specific spectral signature. The grey shaded area around the spectra visualizes the standard deviations of the mean spectra of replicate measurements.

Since the scope of the present study was to use Raman microspectroscopy for the identification and differentiation of vaccine products, our initial analysis was focused on the investigation of technical replicates to determine whether the preparation and background noise could represent a limitation as observed in many high-throughput biological experiments.

### Technical reasons for variance of Raman spectra

**Fig. 3:**
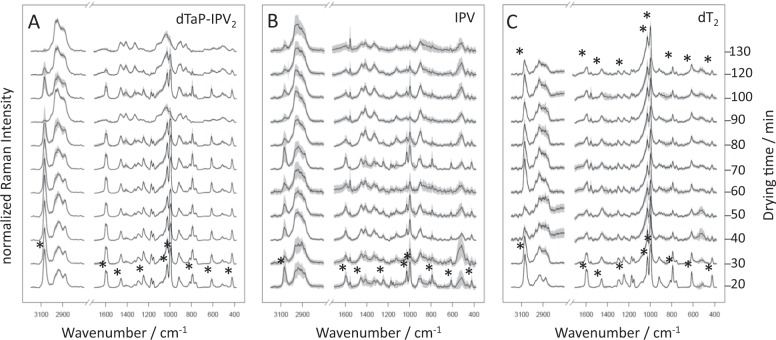
Technical variance of Raman spectra depending on the length of drying. For a detailed view, the Raman data of the three vaccine products dTaP-IPV_2_, IPV, dT_2_ were split into the data subsets of the replicate measurements. The mean spectra of the separate technical replicates are plotted according to drying time (from bottom to top). The standard deviations are visualized by the grey shades around each spectrum. Prominent signals of phenoxyethanol are highlighted by asterisks in analogy to Badawi et al.^19^. The signal intensities of phenoxyethanol decrease to different extents during the course of drying. All spectra were normalized for an effective comparison and shifted vertically for visualization

While examining air-dried vaccine samples by means of Raman microspectroscopy two sources of technical variance were identified. First, the drying procedure of the colloidal vaccine droplets caused a spatial inhomogeneity of the vaccine material during evaporation, which is known as the coffee-ring effect.^18^ Therefore, concentration gradients within the air-dried droplet resulted in spatial inhomogeneities and therefore in variation of Raman signal intensity at different grid points (Fig. 1 3D data cube, Fig. S1). Second, vaccine products containing phenoxyethanol (Table 1) showed variations within their Raman signature that roughly correlated with the drying time. In Fig. 3a–c the mean spectra of dTaP-IPV_2_, IPV, and dT_2_ are depicted. Each spectrum summarizes the Raman signatures of one replicate mapped after the indicated drying interval. The mean spectra of the first replicates were measured after a drying interval of 20 min. These spectra were dominated by Raman signals (highlighted by asterisks) that were previously assigned to phenoxyethanol by Badawi et al.^19^. These signals diminished in intensity the longer the vaccine suspensions were dried at room temperature, albeit to varying extents: For the vaccine products dTaP-IPV_2_ and IPV the signals disappear almost completely after a drying period of 2 h (Fig. 3a, b) while in dT_2_ the phenoxyethanol signals remain prominent in the Raman signature within the observed time span. Furthermore, the dried spots of a vaccine suspension are not identical preparations, they rather represent technical replicates. Therefore, slight differences in the drying behaviour were obtained in the microscope images of distinct vaccine spots (Fig. S1). Also the evaporation behaviour of the phenoxyethanol is subjected to such fluctuations, Fig. 3a. We suggest, each replicate has its individual drying kinetic. Therefore, the phenoxyethanol Raman signals can still be present after 100 min drying time in one spot while they were disappeared after 90 min drying in another spot (Fig. 3a). Aside from such fluctuations, the trend of the decreasing Raman signal intensities of phenoxyethanol with ongoing drying time due to evaporation is evident in the mean spectra of Fig. 3a, b. For the statistical modelling, it is important to be aware of such variations and subsequent to include them for a robust training data set.

**Table 1:** Vaccine products used in this study

Vaccine product (antigen composition)	Adjuvant	Phenoxyethanol	Manufacturer
T_1_	Al(OH)_3_	No	A
T_2_	Al(OH)_3_	No	C
dT_1_	Al(OH)_3_	No	C
dT_2_	AlPO_4_	Yes	A
dTaP	Al(OH)_3_+AlPO_4_	No	B
dTaP-IPV_1_	Al(OH)_3_+AlPO_4_	No	B
dTaP-IPV_2_	AlPO_4_	Yes	A
dT-IPV	Al(OH)_3_	Yes	A
IPV	none	Yes	A
DTaP-IPV-HepB	Al(OH)_3_+AlPO_4_	No	B
HepB	Al(OH)_3_	No	B

Vaccine antigens: *T* tetanus, *d* diphtheria (low antigen content), *D* diphtheria (high antigen content), *aP* acellular pertussis antigens, *IPV* inactivated poliovirus, *HepB* hepatitis B

### Data evaluation by linear discrimination analysis (LDA)

The aim of the statistical analysis of the vaccine Raman data is to find the discriminating features in the spectral data and model the differences between the vaccine products so that unknown samples could be identified by their Raman signature. Applying linear discrimination analysis (LDA), the statistical properties of the particular vaccine types were calculated. To correct for the spatial inhomogeneities in the mapping area of the vaccine material, single Raman spectra of one map were summarized to subsets of averaged spectra by a randomized averaging procedure. The procedure is visualized in the right part of Fig. 1. Ten spectra from the 2D data matrix were successively picked without replacement and averaged. Therefore, the data set of each replicate was reduced from the original 100 to 10 spectra. The reduced data matrices (Fig. 1) were utilized for statistical modelling. To consider the vaccine formulation dependent evaporation behaviour of the phenoxyethanol, Raman data from replicates recorded after different drying times were included into the present vaccine data set.

**Fig. 4:**
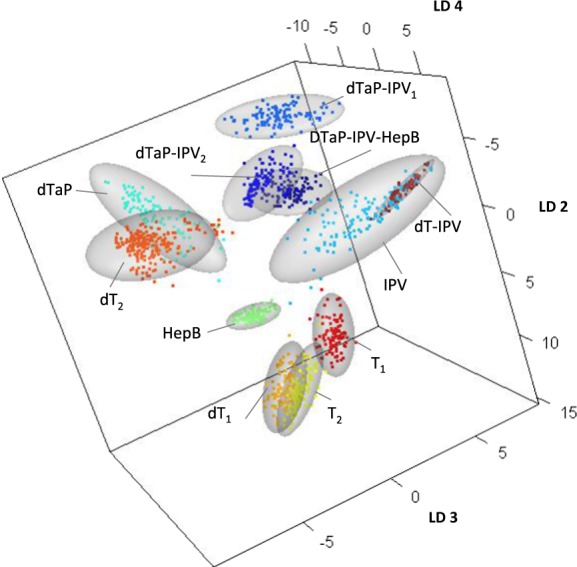
Classification of the vaccine products are represented by the discriminant functions LD2, LD3 and LD4 in a 3D scatterplot. Raman data derived from the replicate measurements of the particular vaccine products are represented by colour-coded squares, ellipsoids visualize the 95% confidence interval for each type. Vaccine Raman data cluster according to their Raman spectral similarities close together or in distinct areas of the spectral feature space

In Fig. 4a the 3D scatterplot of the discriminant functions (LD2–LD4) are plotted to visualize one discrimination possibility between vaccine Raman data. The Raman data derived from the replicate measurements of the particular vaccine types are represented by colour-coded squares. The ellipsoids visualize the 95% confidence interval for the tested batches of each product. The scatterplots reveal that the vaccine Raman data cluster according to their Raman spectral similarities close together or in distinct areas of the spectral feature space. The sizes of the ellipsoids and the spatial spread of the data points visualize the variance of the data within each type and therefore the variance of the technical replicates.

### Discrimination of similar vaccine formulations

**Fig. 5:**
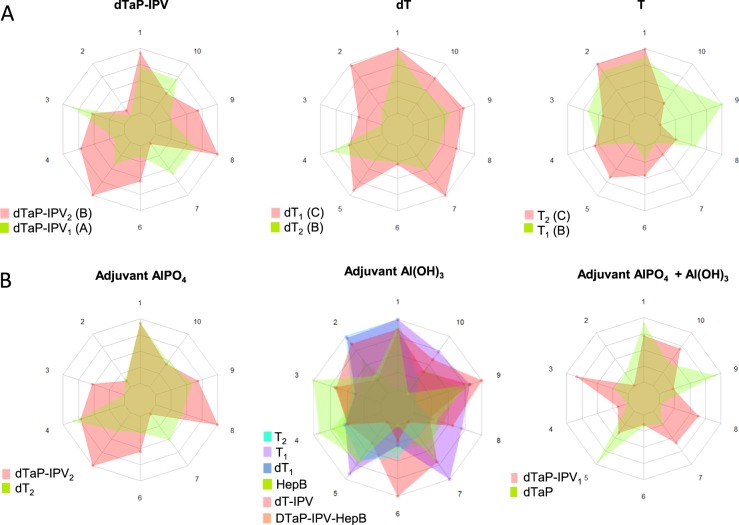
Discrimination of similar vaccine formulations. The radar plot provides information on the statistical properties of the vaccine model. The predicted scores for each LD function are depicted. Big differences between the LD values of the compared vaccine products in one particular direction reveal big spectral differences. **a** Analysis of vaccine products containing the same composition of active substances. Vaccine products containing the same set of vaccine antigens display differences in their Raman spectra that can be exploited for product identification. **b** Comparison of vaccine products with similar adjuvants. Subtle differences derived from the active substances can be recognized in the Raman spectra of vaccines containing the same adjuvant

The performance of the classifier for vaccines with equivalent formulations was investigated and the results are shown in Fig. 5. The radar plot provides information on the statistical properties of the vaccine model at a glance. Big differences between the LD values of the compared vaccine products in one particular direction reveal big spectral differences in the Raman data. Consequently, the smaller the overlapping surfaces in the radar plot become the more distinct the differences of the Raman signatures of the compared vaccines are. Figure 5a depicts the comparison of the vaccine products containing the same vaccine antigens but produced by different manufacturers. Both dTaP-IV vaccine products differ in their Raman spectral profiles shown in Fig. 2b, which becomes evident in the small surface overlap in the radar plot. Also, both dT vaccine products differ in their Raman spectral profile (Fig. 2a). The larger surface area of dT_1_ when compared to dT_2_ indicates a clear statistical difference of the spectral features. The Raman signatures of both tetanus vaccines (T_1_ and T_2_) in Fig. 2a look very similar but the radar plot of the tetanus vaccines in Fig. 5a reveals a clear offset of the surface areas and therefore clearly distinguishable Raman data. Similarly, vaccine products containing the same adjuvants were compared in Fig. 5b. The offsets of the surface areas indicate, that the differences between the respective vaccine products are clearly reflected by the spectroscopic data.

### Optimization of LDA modelling

Classification rates were estimated using a leave-one-replicate-out cross-validation procedure to ensure the ability of the approach to work in routine laboratories. The vaccine model was tested by leave-one-replicate-out cross-validation meaning that a LDA model was constructed based on training data that included all but one experimental replicate, while the left-out replicate served as validation data for testing. This was performed once for all replicates of all vaccine products. The prediction result for one measured replicate (technically independent Raman maps) was assembled by the prediction result of its ten pre-arranged spectra (Fig. 1). If the prediction result was not uniform for spectra of one replicate, the prediction output was based on majority rule voting. The results for all vaccines are summarized in Table 2. In the diagonal, the percentage of correctly predicted replicates is shown. The sensitivities and specificities for each vaccine type are indicated below the table, together with the number of measured replicates and the number of averaged spectra included in the statistical analysis. The numbers of the original Raman spectra derived from the mapping measurements are indicated in the last row of the table.

## Discussion

The present work demonstrates the potential of the Raman spectroscopy to analyse and classify complex manufacturer-specific vaccine formulations with one single analytical method. The methodological approach presented in this study provides a good basis for development of an easy and reliable system for vaccine product identification based on a vaccine’s inherent Raman spectral features. The fingerprint-like specificity of a vaccine product’s spectral signatures observed in this study could be exploited to setup a reference database of well-standardized Raman spectra of vaccine products to estimate batch-to-batch variation. Thus, this study addresses an unmet need for a cost-effective, label-free and reliable screening method with minimal preparation and measurement effort in vaccine control.

The examination of dried fluids containing proteins in submicromolar concentrations by the Raman spectroscopy is a well-established and straightforward approach, known as drop coat deposition Raman spectroscopy (DCDRS).^20–22^ Here, we combined the spectral analysis of thin layers of air-dried vaccine solution with statistical modelling for vaccine product identification.

The drying of 1 µl of vaccine sample permitted us to record a reproducible and representative spectral fingerprint of a vaccine. The data management approach used corrects for spatial inhomogeneities in the dried sample while offering a proper representation of the original sample’s inherent chemical signature. To equalize spatial inhomogeneities caused by local bulky spots or areas with less vaccine substance within the air-dried spot, a randomized averaging process was introduced to the data of the Raman maps. Subsequently, the product-specific spectral features sampled from spatially distributed vaccine material were summed up and intensified while infrequently appearing artefacts were averaged out. The randomized averaging procedure of the mapping data makes the introduced approach reproducible and more robust against technical variations.

Because, a Raman spectrum is the sum of the Raman signals of all chemical components within a sample, the information on multiple vaccine components and the excipients is probed almost instantaneously. The pattern of spectroscopic variations (i.e., changes in peak heights, widths, positions, relative heights and widths, etc.) reflects the pattern of variation within the vaccine formulations, which consist of a variable number of different components. However, a spectrum with more than 1000 wavenumbers does not contain a thousand pieces of independent information. The resulting spectrum is formed by the superposition and the reciprocal influence of the underlying spectral values simultaneously recorded from all chemical constituents, e.g., vaccine antigens, adjuvants, buffer components and other excipients. These signals are further influenced by the presence of solvents, the pH and physical properties, such as formation of crystals or amorphous particles within the vaccine suspension. Thus, the mixture itself and other interfering effects complicate the analysis of the spectral data. Well-established computational methods were applied to correct for the influence of the instrumental setup or background noise.^23,24^

A common method for dimension reduction is the principal component analysis (PCA).^25,26^ PCA transforms a set of possibly correlated response variables into a new set of non-correlated variables, referred to as principal components (PC). The output of the PCA is the components in the order of significance. Components with less significance (assigned to noise) can be ignored. Therefore, the dimension of the data is reduced without loss of information.^27^ Subsequently, the data were classified by LDA to capture the particular combination of spectral values that form the unique spectra of defined vaccine formulations. The output of this analysis was a vaccine product-specific LD value that could be utilized as a product-specific identifier once systematic batch-to-batch analysis has been carried out.

Notably, classification rates were estimated using a leave-one-replicate-out cross-validation procedure to ensure the applicability of the approach in routine laboratories. The classification rules defined for the LD value can subsequently be applied for the allocation of new and unknown samples. Reproducibility and robustness of the model was evaluated by a leave-one-replicate-out cross-validation.^28^

In combination with batch-to-batch variances technical variability can hamper the statistical analysis of the data, thus, increasing the risk for misinterpreted results. Thus, the technical variance information is a crucial point to evaluate the robustness and sensitivity in downstream data analysis.^29^ Technical reproducibility for the Raman spectroscopic analysis of the air-dried vaccine suspension was demonstrated to be effective within a time frame of 2 h. The present study revealed that a proper adaption of the drying intervals for different vaccine products containing the volatile excipient phenoxyethanol considerably increases the classification accuracy. For example, with longer drying intervals and subsequent decreasing phenoxyethanol signal intensities, the misclassification between dT_2_ for dTaP-IPV_2_ was reduced. Nevertheless, shorter drying intervals were beneficial to differentiate the investigated IPV product from the dT-IPV vaccine. Our data suggest that for Raman measurement of vaccines containing phenoxyethanol a time series of different drying intervals with at least three time points (including one droplet preparation with a drying period of 20 min, 2 h and one time point in-between, respectively) should be recorded for an optimal result.

Notably, phenoxyethanol is used as preservative in some vaccine products.^30^ It is a highly volatile solvent and its drying kinetic within a complex vaccine formulation depends on the present molecular interactions. In forensic science, determining the phenoxyethanol concentrations on an ink stroke on paper is a common method to trace the course of drying.^31,32^ The data presented suggest that, similarly, the observed phenoxyethanol evaporation kinetic is a specific feature of the vaccine formulation.

Another characteristic feature of the DTaP vaccines is that vaccine antigens are typically adsorbed to adjuvants, such as Al(OH)_3_ and AlPO_4_.^14^ The results of the present study reveal that despite the signals derived from the same adjuvant in different vaccine products the sensitivity of the Raman technology is high enough to allow the detection of subtle differences in spectra that arise from the specific vaccine formulation, e.g., the combination of antigenic components and adjuvant (Fig. 4b).

The potential confusion of T_2_ and dT_1_ was due to very similar spectral profiles, which could not be explained by spectral changes during the course of drying. A possible explanation could be that T_2_ and dT_1_ are subject to similar manufacturing processes that result in specific excipients or other residuals causing the specific signal overlap in the present vaccine data set. However, differences in the Raman data of these vaccines were captured and can be modelled by LDA. It is, thus, expected that with an increasing sample size the differentiation between T_2_ and dT_1_ Raman spectra can be highly improved. Nevertheless, in the present study the possible confusion is limited to these two specific vaccine products; thus, the error rates caused by T_2_ and dT_1_ did not question the classification of other vaccines and will not lead to the assignment of false positives or false negatives within the remaining vaccines. In this context it should further be noted that the distinction of vaccines with similar vaccine antigen composition and identical clinical indication, such as T_1_ and T_2_ or dT_1_ and dT_2_ were not mistaken for one another (Table 2). Thus, the results of the analysis demonstrate that Raman spectroscopy is an appropriate and reliable analytical tool for the differentiation of vaccines of similar composition. The signatures obtained can be exploited for product identification.

**Table 2:** Confusion table of the cross-validation. Classification rates were estimated using a hold-out validation procedure to ensure applicability in routine laboratories.

	True label [%]	T_1_	T_2_	dT_1_	dT_2_	dTaP	dTaP-IPV_1_	dTaP-IPV_2_	dT-IPV	IPV	DTaP-IPV-HepB	HepB
Predicted vaccine label [%]	T_1_	**100**	0	0	0	0	0	0	0	0	0	0
	T_2_	0	**90**	20	0	0	0	0	0	0	0	0
	dT_1_	0	10	**80**	0	0	0	0	0	0	0	0
	dT_2_	0	0	0	**95**	0	0	0	0	0	0	0
	dTaP	0	0	0	0	**100**	0	0	0	0	0	0
	dTaP-IPV_1_	0	0	0	0	0	**100**	0	0	0	0	0
	dTaP-IPV_2_	0	0	0	5	0	0	**100**	0	0	0	0
	dT-IPV	0	0	0	0	0	0	0	**100**	9	0	0
	IPV	0	0	0	0	0	0	0	0	**91**	0	0
	DTaP-IPV-HepB	0	0	0	0	0	0	0	0	0	**100**	0
	HepB	0	0	0	0	0	0	0	0	0	0	**100**
	Sensitivity [%]	100	90	80	95	100	100	100	100	91	100	100
	Specificity [%]	100	98	99	100	100	100	99	99	100	100	100
	Number of replicates	10	10	10	21	10	11	11	10	11	10	10
	Number of averaged spectra	100	100	100	210	100	110	110	100	110	100	100
	Number of original spectra	1000	1000	1000	2100	1000	1100	1100	1000	1100	1000	1000

The table-diagonal shows the percentage of correctly predicted replicates

The high sensitivities and specificities for vaccine identification found in this study highlight that Raman spectroscopy could serve as a reliable and robust analytical tool delivering few false negative or false positive results. This was especially evident for DTaP vaccine products that displayed validated classification accuracies of 100%. The combination of machine learning and statistical modelling provides unbiased product identification based on the Raman spectra of the final vaccine product. However, the degree of batch-to-batch variation for each vaccine was not systematically investigated in this study.

Since the data indicated that the Raman signature of the individual product is unique, we refrained from an in-depth analysis of the Raman spectra to identify the underlying chemical components. However, other applications might require this analysis to confirm the presence of specific vaccine antigens and adjuvants. These results could, thus, also pave the way for application of the presented workflow for confirmation of vaccine batch consistency: the easily accessible product- and potentially batch-specific information provided by the introduced technical concept can be entered into product-specific databases. This approach is well in-line with the presently pursued quality control concept based on comparative analysis for consistency testing.^33–35^

## Materials and methods

### Vaccines

Eleven different commercially available vaccine products were tested (Table 1). The vaccines were stored at 2–8 °C until measurement. Unfortunately, some licensed vaccine products could not be included in the study because of unavailability due to supply shortages.

### Sample preparation

For Raman measurements vaccine suspensions were extracted from the containers into Eppendorf tubes, homogenized by vortexing and 1 μl was applied onto a CaF_2_ slide and dried at room temperature. The sample size was predicted using the learning curve^36^ resulting in five replicates to be necessary. Therefore, we planned with 10 in every group to ensure a valid statistical outcome. Twenty minutes after preparation the samples were visibly dry and the measures started with the first replicate. The last replicate was measured after ~2 h later.

### Raman measurements

The air-dried vaccine preparations were mapped by grid defined Raman measurements. A 1000 × 1000 µm sample raster with 10 × 10 measurement points were arranged per droplet. Accordingly 100 spectra were recorded for each sample. Raman maps were recorded with a WITec UATS 300 spectrometer (Ulm, Germany), combined with a Zeiss microscope equipped with a Zeiss EC EPIPLAN objective (20×/0.4). The beam of a diode laser with an excitation wavelength of 514 nm was focused with a power of ~50 mW onto the sample. A piezo-electrically driven scanning stage moved the sample through the laser focus in a raster pattern. The step size between two raster points was set to 100 µm. For each measurement position one spectrum was recorded with an acquisition time of 5 s. The back scattered light was collected (through the same microscope objective), diffracted using a 600 lines/mm grating with a spectral resolution of 5 cm^−1^ and detected with a Peltier cooled electron-multiplying charge-coupled device (EM-CCD, −65 °C) camera.

### Data pre-processing

The statistical language R was applied for chemometrical analysis and computations (R).^37^ The workflow of the data pre-processing was previously described.^38^ In summary, a cosmic spike removal followed by wavenumber calibration with 4-acetaminophenol^39^ background correction with the SNIP algorithm and finally a vector normalization of each Raman spectrum was carried out.

Each Raman map of a technical independent replicate represents a spectral data cube containing 100 single spectra. Spectra of all pixels were sequentially arranged in a 2D data matrix. To obtain a comprehensive Raman spectral signature of a specific vaccine product, the 2D data matrices derived from the replicate measurements were summarized by averaging. For statistics, a correction for the spatial inhomogeneities was required. Therefore, the 2D data table of each replicate was reduced from the 100 original spectra to 10 representative spectra applying an averaging procedure. For this purpose, 10 randomly chosen raster points were successive picked without replacement and averaged. The 10 mean Raman spectra were utilized to construct a reduced data matrix and these matrices were utilized for statistical modelling.

### Statistical modelling

As classification model a combination of principal component analysis (PCA) and linear discriminant analysis (LDA) was applied to model the differences between the vaccine products. A dimension reduction via principal component analysis (PCA) was carried out and the first 12 principle components (PCs) were introduced to the LDA. Applying LDA, the statistical properties of the particular vaccine products were calculated, which includes the mean spectra for each type and the covariance matrix calculated across all types. In doing so, for the 11 vaccine product, 10 discriminative functions were determined by the LDA in the spectral space to classify the vaccine data set. The LDA model was validated by a leave-one-replicate-out cross-validation.^28^ For this purpose, all Raman spectral data except the data of one replicate were used to build a LDA model and then the model was utilized to predict the left-out subset. This method is repeated so that spectra of each replicate are predicted once. A majority vote was applied to determine a comprehensive prediction for a replicate. This voting scheme was applied to avoid mixed predictions, if the prediction result was not uniform for spectra of one replicate.

The radar plot visualizes the vaccine LDA model in a two-dimensional plot. The ten spokes in each plot visualize the 10 discriminative functions of the model. The LD scores contain the class-discriminatory information. For the radar plots,^40^ the predicted scores for each LD function were min/max normalized to scale the data between 0 and 1. Vaccines with similar score values are localized close together along one spoke line. The bigger the spectral differences are between the respective vaccines, the more different are their score values and the longer is the distance between the scaled scores along one spoke line.

### Code availability

The software “R” and the utilized packages “peaks”, “MASS”, “fields”, “scatterplot3d” and “fmsb” are open source software and freely available. The in-house written procedures for data pre-processing can be obtained from T.B. and J.P. upon reasonable request.

## Electronic supplementary material

Supplementary Figure S1

## Data Availability

All data generated or analysed during this study are included in this published article and its supplementary information files.

## Electronic supplementary material

**Supplementary information** accompanies the paper on the *npj Vaccines* website (10.1038/s41541-018-0088-y).
