# Supplementary material for: Raman spectroscopy-based identification of toxoid vaccine products
Source: NPJ Vaccines. 2018 Oct 4;3:50. doi: 10.1038/s41541-018-0088-y (PMC6172244; doi:10.1038/s41541-018-0088-y)
Supplement: Supplementary file 1 — Supplementary Figure S1 [file 41541_2018_88_MOESM1_ESM.pdf]

Supplementary

Raman spectroscopy-based identification of toxoid vaccine products

Anja Silge[1,2], Thomas Bocklitz[1,2,3], Björn Becker[4], Walter Matheis[4], Juergen Popp[1,2,3], Isabelle Bekeredjian-Ding[4]

[1]Institute of Physical Chemistry and Abbe Center of Photonics, Friedrich-Schiller University Jena, Helmholtzweg 4 D-07743 Jena, Germany

[2]InfectoGnostics Research Campus Jena, Centre of Applied Research, Philosophenweg 7, D-07743 Jena, Germany

[3]Leibniz Institute of Photonic Technology, Albert-Einstein-Str. 9. D-07745 Jena, Germany

[4]Division of Microbiology, Paul-Ehrlich-Institut, Paul-Ehrlichstr. 51-59, D-63225 Langen, Germany

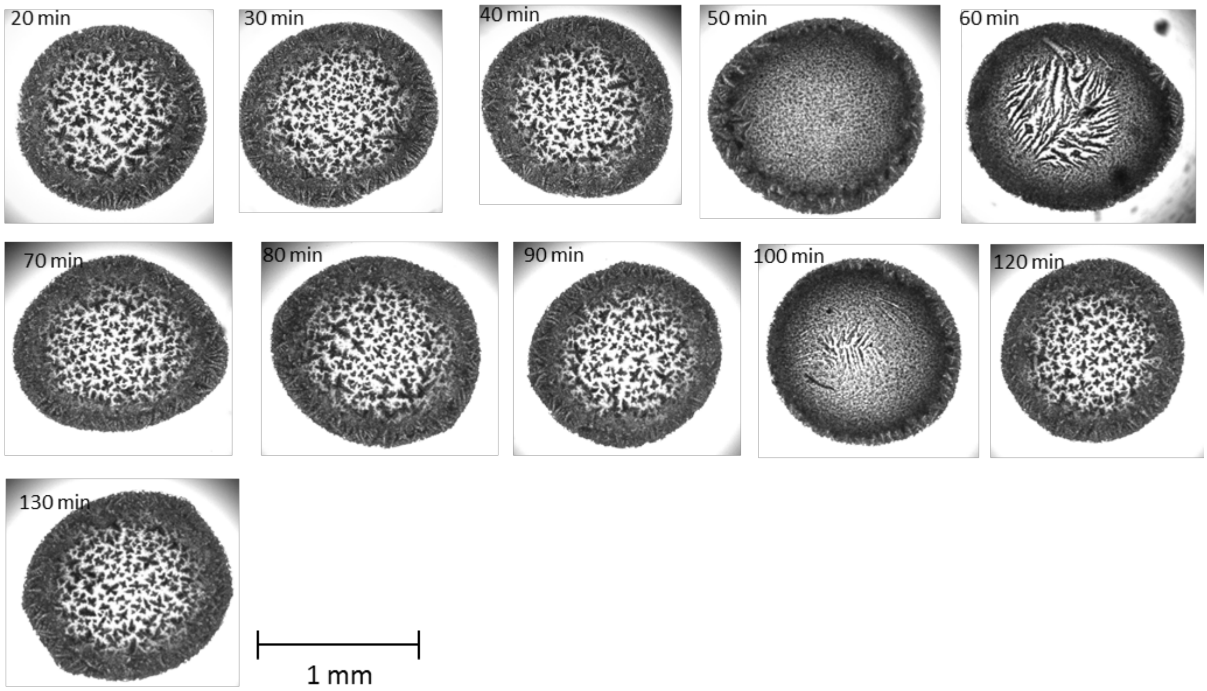

Fig. S1: Spatial inhomogeneity of the vaccine material during evaporation. Microscope images of the 11 replicates prepared for the vaccine dTaP-IPV$_{1.}$ Indicated are the time points of the Raman spectroscopic analysis.
